# circRNAs Signature as Potential Diagnostic and Prognostic Biomarker for Diabetes Mellitus and Related Cardiovascular Complications

**DOI:** 10.3390/cells9030659

**Published:** 2020-03-09

**Authors:** Mohamed Zaiou

**Affiliations:** School of Pharmacy, Institut Jean-Lamour, The University of Lorraine, 7 Avenue de la Foret de Haye, CEDEX BP 90170, 54500 Vandoeuvre les Nancy, France; mohamed.zaiou@univ-lorraine.fr; Tel.: +33-03-72-77-90-15; Fax: +33-0-3-83-68-23-01

**Keywords:** circular RNAs (circRNAs), biomarker, epigenetics, microRNAs (miRNAs), diabetes mellitus, cardiovascular diseases (CVD)

## Abstract

Circular RNAs (circRNAs) belong to the ever-growing class of naturally occurring noncoding RNAs (ncRNAs) molecules. Unlike linear RNA, circRNAs are covalently closed transcripts mostly generated from precursor-mRNA by a non-canonical event called back-splicing. They are highly stable, evolutionarily conserved, and widely distributed in eukaryotes. Some circRNAs are believed to fulfill a variety of functions inside the cell mainly by acting as microRNAs (miRNAs) or RNA-binding proteins (RBPs) sponges. Furthermore, mounting evidence suggests that the misregulation of circRNAs is among the first alterations in various metabolic disorders including obesity, hypertension, and cardiovascular diseases. More recent research has revealed that circRNAs also play a substantial role in the pathogenesis of diabetes mellitus (DM) and related vascular complications. These findings have added a new layer of complexity to our understanding of DM and underscored the need to reexamine the molecular pathways that lead to this disorder in the context of epigenetics and circRNA regulatory mechanisms. Here, I review current knowledge about circRNAs dysregulation in diabetes and describe their potential role as innovative biomarkers to predict diabetes-related cardiovascular (CV) events. Finally, I discuss some of the actual limitations to the promise of these RNA transcripts as emerging therapeutics and provide recommendations for future research on circRNA-based medicine.

## 1. Introduction

Diabetes mellitus (DM) is a very common complex of endocrine and metabolic disorder. This chronic disease is characterized by the presence of chronic hyperglycemia resulting from defects in insulin secretion, action, peripheral insulin resistance, or all of them [1]. The worldwide and fast-growing epidemic of diabetes is expected to be the leading cause of death in the next decades due to the population ageing and dramatic lifestyle changes. Following the estimate from The International Diabetes Federation in 2017, about 424.9 million people aged 20–79 years lived with diabetes and this number is expected to increase by 63% by 2045 [2]. Moreover, the latest research shows that diabetic patients with chronic hyperglycemia are more likely to suffer from many life-limiting and life-threatening macrovascular cardiovascular diseases (CVD) [3].

There are several types of DM and the two common forms are type 1 diabetes mellitus (T1DM) and type 2 diabetes mellitus (T2DM). T1DM is the result of a chronic inflammatory process that is thought to be mediated by an autoimmune attack resulting in the destruction of pancreatic β-cells causing low or no insulin production. T2DM is the most complex and common form in the general population accounting for >90% of all diabetes. In addition to these two forms, there are other types of diabetes, which are due to specific genetic defects, metabolic and mitochondrial abnormalities, and additional conditions that impair glucose tolerance [4]. However, diabetes could be more complex than we think as a recent study carried out on a Scandinavian population reported that 5 sub-types or clusters of diabetes may exist [5]. In this observational follow-up study, the authors revealed potentially clinically important differences in disease progression and risk of complications between the clusters [5]. Cluster 1 corresponds to what could be called classic T1DM, while the other four clusters correspond to type 2-like diabetes at diagnosis. Cluster 2 corresponds to severe insulin deficient diabetes with low fasting c-peptides and high HbA1c. Cluster 3 shows severe insulin resistant diabetes with high fasting c-peptides and high HbA1c. Cluster 4 corresponds to mild obesity-related diabetes with high BMI and relatively low HbA1c at diagnosis. Finally, cluster 5, or mild age-related diabetes, is characterized by higher age at diagnosis and relatively low AbA1c [5].

Early investigations using different experimental models revealed that diabetes arises through a very complex interplay between genetics, lifestyle, immunological, and environment factors [6,7]. Despite intense research efforts in the field, which have undoubtedly increased our knowledge about disease mechanisms and treatment modalities, the exact etiopathogenesis of diabetes remains elusive and the number of diabetes cases and associated mortality continue to grow worldwide. Hence, there clearly remains an unmet clinical need to develop novel predictive biomarkers as they may help early diagnosis and recognition of diabetes along with the identification of novel therapeutic targets.

In the last decades, several groundbreaking studies have focused attention on epigenetic mechanisms and their potential implication in diabetes. Epigenetics or epigenomics refers to all the alterations and ensuing phenotypes such as changes in DNA conformation, transcription, or translation, that do not involve changes to the underlying DNA sequence [8]. There are multiple levels of epigenetic regulation including DNA cytosine methylation, histones post-translational modifications in chromatin and lately noncoding RNAs (ncRNAs) such as long noncoding RNAs (lncRNAs), microRNAs (miRNAs) and circular RNAs (circRNAs). Over the last few years, ncRNAs have moved from being dismissed as artifacts to be considered as key epigenetic regulators of multiple cellular processes [9]. In this sense, huge efforts have been made since to understand the dysregulation of lncRNAs and miRNAs under diabetic conditions [10,11]. More recently, researchers have shifted their research focus to circRNAs hoping to develop these molecules as new biomarkers for early detection and management of diabetes. Herein, I will summarize the current understanding of the emerging roles of circRNAs in the pathophysiology of diabetes and address their potential to be therapeutic targets to alleviate the clinical burden of diabetic vasculopathy.

## 2. General Characteristics of circRNAs

### 2.1. circRNA Properties

Recent methodology of high-throughput sequencing and computational algorithms has enabled the identification of a variety of endogenous circRNAs in different tissues. These RNA species are usually derived from precursor mRNA back-splicing, a molecular event in which a downstream 5′ splice site is joined to a downstream 3′ splice site to form a covalently closed circular structure [12], hence named circular RNAs. Surprisingly, circRNAs arise from different regions of expressed genes. With respect to their biogenesis, several models have been proposed, including direct back-splicing with ALU and inverted repeats complementation, exon lariat, and RNA-binding-protein (RBPs) mediated models [13]. However, these are probably not the only mechanisms of circRNAs biogenesis as how a region of the transcriptome can circularize and under which condition this happens remain elusive. For ample details on the regulation of circRNAs biogenesis, I refer the reader to excellent reviews published recently [13,14,15]. Advances in RNA sequencing and recent bioinformatics tools have resulted in the discovery and annotation of thousands of circRNAs based on their genomic location. They have been classified into at least three types with distinct regulatory functions in the mammalian cell: circular RNAs derived from exons (EcircRNAs), circRNAs derived from lariat introns (ciRNAs), and circRNAs derived from exons with retained introns (EIciRNAs) [12,14]. Furthermore, several studies have shown that circRNAs are exceptionally stable evolutionary conserved among species, diverse, and often show tissue or development stage-specific expression pattern [16,17] pointing to their potential diverse cellular functions in various biological processes [17,18].

### 2.2. circRNAs Potential Functions

Although all biological functions of circRNAs are not entirely discovered, some are well described in the literature. Evidence has suggested that a subset of circRNAs can act as decoys or sponges for specific miRNAs, thereby upregulating or downregulating miRNAs target genes expression [19,20]. Other studies reported that circRNAs may compete with mRNAs for binding miRNAs in the cytoplasm and thus contribute to fine-tuning gene expression at the post-transcriptional level [17,19,21]. There are examples in the literature illustrating this process and the two perhaps most representative circRNAs to support this observation are ciRS-7/CDR1as (sponge for miR-7) and mouse SRY (sponge miR-138) [17,19]. However, it was reported that circRNAs acting as miRNA sponges are rare [22] and not all circRNAs might act as miRNAs sponge because a large fraction of these contains fewer mRNAs binding sites than co-linear mRNA transcripts [23]. Furthermore, it is still not known whether circRNAs-miRNAs interaction is only for sequestration of miRNAs or goes beyond this function. Nevertheless, if the sponging capacity of circRNAs turns out to be true, it will have important clinical applications in various pathologies for targeting disease related miRNAs. Thus, more functional studies and biological validation experiments are required to uncover the potential impact of circRNAs-miRNAs interplay on miRNAs target genes expression.

Beyond regulating gene expression by acting via miRNAs, circRNAs may also function as sponges for RNA-binding proteins (RBPs) serving as post-transcriptional regulators of gene expression [23]. For instance, circRNAs have been shown to interact with Argonaute proteins (AGO) of the RNA-induced silencing complex (RISC) that regulates miRNA action [17,24]. Another well supported experimentally example of a circRNA protein sponge is the splicing factor muscleblind (MBL/MBNL1) in *Drosophila* [25]. circPABPN1 also can prevent nuclear poly (A) binding protein 1 (PABPN1) mRNA translation into protein by sponging the RBP Human-antigen R (HUR) in human cells [26]. Moreover, other studies revealed that circular antisense non-coding RNA in the INK4 locus (circANRIL) binds to the c-terminal domain of pescadillo homolog 1, an essential 60S-preribosomal assembly factor, thereby impairing exonuclease-mediated pre-rRNA processing and ribosome biogenesis in vascular smooth muscle cells and macrophages [27]. Taken together, these findings suggest the potential role of the circRNA-protein interaction in regulating different aspects of disease processes.

Although the majority of circRNAs can be found in the cytoplasm, a fraction of these species is transported to the nucleus where it can accumulate and be functional by enhancing the expression of their parental gene in some circumstances [28]. Members of nuclear EIciRNAs [28] and ciRNAs [29] have been shown to interact with transcription machinery and regulate a full spectrum of gene expression. As best-known examples, the circRNAs ci-ankrd52 and ci-sirt-7 were able to enhance their parental genes expression, ankyrin repeat domain 52 (*ANKRD52*) and *RIRT7*, respectively, through interaction with RNA polymerase II elongation complex [29,30]. Additionally, the circRNAs eukaryotic translation initiation factor 3J circRNA (CircEIF3J) and poly(A)-binding protein-interacting protein 2 circRNA (CircPAIP2) have also been found to interact with the U1 small nuclear ribonucleoprotein (snRNP) to form an EIciRNA-U1 snRNP complex that binds RNA polymerase II and fine-tune the transcription of their parental genes [12,28].

circRNAs could have additional functions. They may serve as scaffolds in the assembly of protein complexes. Du et al. demonstrated that circ-Foxo3 can combine with cyclin dependent kinase 2 (CDK2) and p21 (CDKN 1a) to form a ternary RNA-protein complex that represses the function of CDK2 [31]. Recent studies have shown that several of circRNAs can be translated into small peptides in cells [32] and have key roles despite their sometimes-low level of expression [33,34]. In this respect, CircCode (freely available at https://github.com/PSSUN/CircCode) was developed recently as a tool to predict circRNAs with translation potential [35]. Although thousands of circRNAs have been described in humans, only a minor fraction of these shows potentially important biological roles [12,13,17] and a unified explanation for the potential function of the vast majority of circRNAs is still lacking. 

### 2.3. CircRNA Online Resources

To facilitate the study of different aspects of circRNAs, several laboratories have developed various algorithms [14,27,36] to detect circRNA sequences from RNA sequencing data based on different approaches including back-splice junctions. For accurate circRNAs quantification and differential gene expression, Zhang et al. have recently developed a novel algorithm named CIRIquant [37]. Additionally, Chen and colleagues have lately developed an interesting algorithm; circMeta, that can be used for genomic feature annotation and differential expression analysis of circRNAs [38]. These tools have helped to construct numerous circRNA databases (Table 1) with searching and browsing functions. Such searchable databases are made freely by several web servers and provide valuable information of circRNAs (Table 1) [39]. Thus, researchers may need to consult these resources before even launching a project on circRNAs as they may help them in better designing their experiments and making the choice of an adequate study model. However, as RNA technologies advance steadily, more specific versions of databases will merge, which will contain valuable information about the molecular mechanisms and biological roles of circRNAs.

## 3. Insights into the Pathophysiological Role of circRNAs in Diabetes and Related Complications

It is admitted that endothelial dysfunction is a key player and early event in the pathogenesis of vascular complications in DM. The contributing factors underlying impairment of endothelial function are many and commonly include hyperglycemia, insulin resistance, free fatty acid release and lipotoxicity, dyslipidemia, and oxidative stress. Such factors can trigger the pathogenesis of diabetes-associated vascular complications such as diabetic retinopathy, nephropathy, cardiomyopathy and other vascular diseases. Ttiology of diabetes is likely multifactorial and involves interplay between genetic and environmental [6]. Moreover, progressive understanding of its molecular mechanisms has suggested that epigenetics may be and additional layer that controls gene expression and provides a molecular link between genetics and environmental effects on diabetes.

A growing body of literature implicates the dysregulation of ncRNAs in the pathogenesis of diabetes and associated long-term vascular disorders [40]. Altered expression of lncRNAs has been associated with poor glycemic control, insulin resistance, accelerated cellular senescence, and inflammation in diabetes patients [41]. For instance, increased expression of the lncRNA growth arrest-specific 5 (GAS5) and lncRNA ENST00000550337 has been reported in diabetes condition implying that these molecules can serve as a potential diagnostic biomarker for pre-diabetes and T2DM [42,43]. Likewise, lessons from genetic loss- and gain-of-function studies have implicated several miRNAs in the regulation of pancreatic β-cells [44,45]. Aside from lncRNAs and miRNAs, circRNAs have also been demonstrated to play a role in metabolic disorders including diabetes [46,47]. However, these RNA species remain the least understood and appear to have a complex regulatory impact in the pathogenesis of cardiometabolic diseases. In the next section, I will highlight current evidence through which circRNAs exert control of gene expression in the pathogenesis of different forms of diabetes. circRNAs that are most likely to be implicated in diabetes and related vascular disorders are shown in (Table 2).

### 3.1. circRNAs in Hyperglycemia-induced Endothelial Cell Dysfunction

Over the last years, efforts have been made to understand the role of circRNAs in the field of diabetes research. For instance, in human umbilical vein endothelial cells (HUVECs) exposed to high glucose, Shang et al. identified as many as 95 circRNAs that were differentially expressed in the hyperglycemic group and circular RNA NC_000017.11:32358503_32447046 had the highest interaction scores with several miRNAs including hsa-miR-619-5p [48]. Another study revealed that hsa_circ_0054633 regulates high glucose-induced human vascular endothelial cell dysfunction [49]. In a subsequent analysis, the same group reported that hsa_circ_0054633 has a protective effect against high glucose-induced endothelial cell dysfunction through the miR-218/heme oxygenase-1 axes. These basic findings are consistent with a previous research, which has found has_circ_0054633 to be differentially expressed in peripheral blood of patients with T2DM and healthy control [50]. Hence, hsa-circ-0054633 may be involved in the pathogenesis of diabetes and could be used as a biomarker for the diagnosis of T2DM.

Vascular smooth muscle cells (VSMCs) represent another cell model relevant to the biochemical characterization of CVD. A previous study provided evidence that high glucose treatment can induce VSMC proliferation, which is partly mediated by high glucose-activated STAT3/Pim-1 signaling [51]. By performing microarray, Cheng and colleagues found *circWDR77* (hsa_circ_0013509) to be the most significantly upregulated transcript in human VSMCs cultured in vitro under high glucose treatment compared to control cells [52]. These preliminary studies suggest that dysregulated circRNAs may act as novel contributors to the impairment of vascular endothelial cells and therefore to diabetes-related complications. However, definitive evidence to support this hypothesis is still needed.

### 3.2. CircRNAs Regulate Insulin Secretion and β-cells Function

Pancreatic β-cells are a source of insulin production and secretion and the secreted insulin is essential for maintaining homeostasis of blood glucose levels under normal condition. However, when β-cells are altered by various mechanisms, they may lose their identity and, therefore, their functional capacity to control insulin production, which leads to a chronic hyperglycemia and development of DM [53]. Thus, elucidation of the mechanisms involved in insulin regulation and β-cells dysfunction may open additional avenues for the development of novel therapeutic strategies for the treatment of diabetes.

Even though the class of circRNAs has been shown to play a significant role in the development of DM [54,55], very little is known about their role in β-cells function. So far, the best known endogenous circRNA related to diabetes is CDR1as (also termed as ciRS-7) which is generated from a natural antisense transcript of cerebellum degeneration-related antigen 1 (CDR1). Overexpression of CDR1as leads to increased insulin gene transcription and secretion resulting in an overall improved β-cell function [55]. By acting as a powerful miR-7 inhibitor [17], CDR1as promotes islet β-cells proliferation and insulin secretion in diabetes through sponging miR-7 and enhancing Myrip and Pax6 gene expression [55]. Indeed, transgenic mice overexpressing miR-7a in β-cells develop diabetes due to impaired insulin secretion and β-cell dedifferentiation [56]. Furthermore, a recent study by Stoll et al. found that the expression of circHIPK3 and ciRS-7/CDR1as was reduced in the islets of diabetic db/db mice [57]. Silencing these circular transcripts in the islets of wild type animals caused defective insulin secretion, lower β-cell proliferation, and reduced survival, pointing to a possible contribution of altered circHIPK3 and ciRS-7 expression in the development of DM [57]. Similarly, Cao et al. found that the expression of circHIPK3 was significantly decreased in both high glucose-treated HUVECs and in primary aortic endothelial cells (HAECs) from diabetic patients [58]. Accordingly, it can be concluded that ciRS-7 and circHIPK3 might be potential therapeutic targets in diabetes.

### 3.3. Circular RNAs in Diabetes-related Vascular Complications

#### 3.3.1. Diabetic Retinopathy

Diabetic retinopathy (DR) is a severe complication of uncontrolled DM and one of the leading causes of vision loss and blindness [59]. This disease affects approximately one-third of diabetic patients [60] and up to 80% of all patients who have had diabetes for 20 years or more [61]. Hyperglycemia, hypertension, poor glycemic control, dyslipidemia, and longer duration of diabetes are viewed as risk factors and major contributors to microvascular dysfunction occurring in DR [62]. However, the underlying mechanisms of DR are not yet completely uncovered and most patients with DR fail to respond well to currently existing therapeutic modalities.

The role of circRNAs in the pathogenesis of DR has been investigated lately. Gu et al. found that some circRNAs were significantly upregulated in the serum of patients with DR compared to that of controls and diabetes patients without retinopathy [63]. In another study, Zhang and colleagues identified circ_0005015 as the most significantly upregulated circRNA in plasma, vitreous samples, and fibrovascular membranes of diabetic retinopathy patients [64]. Furthermore, siRNA-mediated silencing of circ_0005015 significantly reduced human retinal vascular endothelial cell proliferation, migration, and tube formation. In the same context, Shan et al. reported that a circular non-coding RNA from the HIPK3 gene plays key roles in the development of retinal dysfunctions in diabetes [65]. Interestingly, the authors study showed that in vivo silencing of circHIPK3 attenuated retinal and acellular capillaries, vascular leakage, inflammation, and, therefore, led to improved retinal vascular dysfunction. Mechanistically, circHIPK3 acted as an endogenous miR-30a-3p sponge to increase endothelial cells proliferation and vascular dysfunction [65]. Since cZNF609 was identified as one of the top 10 abundantly expressed circRNAs in endothelial cells [22], a recent study emphasized that overexpression of cirZNF609 increased inflammatory responses by elevating the secretion of IL-6 and TNF-α which aggravated vascular leakage and capillary degeneration in mice model of diabetic retinopathy [66]. In retinal vasculature, cZNF609 was found to be significantly upregulated upon high glucose and hypoxia stress in vivo and in vitro. Further experiments performed by the same authors indicated that cZNF609 silencing decreased retinal vessel loss and suppressed pathological angiogenesis in vivo [66], implying that cZNF609 expression could have diagnostic potential and promising therapy for vascular diseases. More recently, an in vivo study reported that the overexpression of cPWWP2A, a circRNA that acts through miR-579, was able to alleviate diabetes-induced retinal vascular dysfunction [67]. Considering these encouraging preclinical and clinical data, circRNA-based therapy could become a reliable tool in diabetes management.

#### 3.3.2. Diabetic Nephropathy

Diabetic nephropathy (DN), another progressive microvascular complication that develops secondary to diabetes, is one of the most common causes of chronic renal failure in diabetic individuals [68]. As effective treatments for DN are missing, the number of deaths caused by this complication continues to grow. The regulatory roles of ncRNAs in the pathogenesis of DN have received increasing attention. Evidence shows that miRNAs play an important role in the modulation of the renal response to hyperglycemia and the progression of DN [69,70]. However, very little is known about the exact function of circRNAs in the DN condition. One recent study by Hu et al. demonstrated that circRNA_15698 was upregulated in both db/db mice and high glucose-induced mouse mesangial cells [71]. Results from subsequent analysis using bioinformatics tools and luciferase reporter assay proved that circRNA_15698 could act as a sponge of miR-185 to influence the regulation of the transforming growth factor-β1 (TGF-β1). Further, the same study revealed that circRNA_15698/miR-185/TGF-β1 axis could promote extracellular matrix (ECM)-related protein synthesis in diabetic nephropathy progression [71]. Lately, Liu et al. reported that the expression of circ_008045 was positively correlated with the progression of DN and inhibited the cell proliferation and fibrosin in the mesangial cell by sponging miR-24-3p [72].

#### 3.3.3. Gestational Diabetes

Gestational diabetes mellitus (GDM) is a metabolic disorder in pregnancy that is usually accompanied by hyperglycemia, hyperlipidemia, and even high blood pressure. Women with GDM have a markedly higher risk of developing T2DM after delivery [73]. Over the last few years, efforts have been made to understand the role of ncRNAs in the regulation of glucose metabolism in GDM [74]. In this context, miRNAs were dysregulated in the plasma and placenta from women suffering from GDM and associated with pregnancy and birth-related outcomes [75]. Yan et al. reported that circRNAs were aberrantly expressed in the placental villi of the GDM group compared to the control group [76]. By performing microarray, these authors found 482 circRNAs differentially expressed; 227 were increased expression and 255 were downregulated. Further analysis demonstrated that most of these circRNAs harbored miRNA binding sites, and some of these miRNAs were associated with GDM [74,77]. More recently, hsa_circRNA_0054633 was shown to have a closely associated with GDM and a highly diagnostic value for this disorder [78]. Assessment of the expression profile of circRNAs in the placentas of women with GDM revealed that circ_5824, circ_3636 and circ_0395 levels were significantly lower in the GDM group compared to the control group [79]. Taken together, these findings suggest that circRNAs may be associated with the development of GDM and offer a potential for risk prediction and intervention strategies of this disorder, but the underlying mechanisms need further study.

#### 3.3.4. Diabetic Neuropathy

Diabetic neuropathy (DNP) is also one of the most common chronic complications of DM. It is a life-threatening disorder that involves both peripheral and autonomic nerves, affecting more than 50% of the diabetic population [80]. To date, our understanding of the pathophysiology of DNP remains elusive [81]. The role of epigenetics in this disease has been suggested recently. Several reports have shown that ncRNAs including miRNAs and lncRNAs play an important role in the pathogenesis of DNP [82,83]. However, the clinical function of circRNAs in this disease remains partially explored. A recent study reported increased expression of circHIPK3 in serum from diabetic patients who suffered from diabetes neuropathic pain and in dorsal root ganglion from STZ-induced diabetes rats [84]. Interestingly, this study presented evidence that intrathecal circHIPK3 shRNA treatment can be used to treat neuropathic pain of diabetic rats [84]. In another study, Zhou et al. [85] analyzed the expression profile of ncRNAs in relation to that of mRNAs in the spinal cord of the Sprague-Dawley Rats which sustained spared nerve injury (SPI). The authors found a total of 134 lncRNAs, 12 miRNAs, 188 circRNAs, and 1066 mRNAs dysregulated on day 14 post-SNI. More recently, Liu Y et al. [86], measured the impact of the autophagy-related circular RNA (ACR) on rat Schwann RSC96 cells which underwent high glucose irritation, an in vitro model for diabetic peripheral neuropathy. They observed that ACR relieved treated RSC96 cells apoptosis, autophagy, and oxidative stress through miRNA-145-3p downregulation. Nevertheless, further studies are needed to investigate the mechanisms by which circRNAs can be involved in DNP as these may enable a rational approach for the treatment of such a disease.

#### 3.3.5. Diabetic Cardiomyopathy

Diabetic cardiomyopathy (DCM), a major cardiovascular complication of diabetes, is defined as the existence of abnormal cardiac structure and performance in the absence of other cardiac risk factors, such as coronary artery disease (CAD) and hypertension [87]. Several factors including hyperglycemia, insulin resistance, increased fatty acid metabolism, myocardial fibrosis, cardiomyocyte death, and inflammation are shown to contribute to the pathophysiology of DCM [88,89]. However, the molecular mechanisms involved in this pathology remain partially determined. On this subject, several studies conducted in recent years aimed to explore the role of ncRNAs in the pathogenesis of DCM. For instance, upregulation of miRNA-203 was found to inhibit myocardial fibrosis and oxidative stress in mice with diabetic cardiomyopathy [90]. LncRNAs abnormal expression was also reported to be significantly associated with DCM [91]. Indeed, the expression of the lncRNA-myocardial infarction-associated transcript (MIAT) was found to be upregulated in models of diabetic cardiomyopathy and its silencing significantly improved cardiac function and decreased cardiomyocyte apoptosis [92,93].

While great interest and enthusiasm have been aroused in the research area of miRNAs and lncRNAs in DCM, studies concerning circRNAs have fallen behind. In this context, emerging evidence suggests that circRNAs are also implicated in the process of diabetes and DCM. For instance, Tang and colleagues have reported that circRNA_000203 is upregulated in the diabetic mouse myocardium and in angiotensin (Ang) II-induced mouse cardiac fibroblasts [94] and might serve as a potential target for prevention and treatment of cardiac fibrosis in DCM [94]. circRNA_010567 was found to promote myocardial fibrosis through the miR-141/TGF-β1 pathway in DCM [95]. More recently, Yang and colleagues have demonstrated that hsa_circ_0076631, named caspase-1-associated circRNA (CACR), is highly expressed in high-glucose-treated cardiomyocytes and in the serum of diabetic patients [96]. The same study has provided further evidence that hsa_circ_0076631 can target miR-214-3p/caspase-1 pathway to mediate pyroptosis of diabetic cardiomyopathy. Nevertheless, further studies are needed to investigate additional mechanisms by which circRNAs are involved in DCM.

## 4. circRNAs in Diabetes as Useful Biomarkers in the Diagnosis of Cardiovascular Disease States

Currently, a number of evidences exist, demonstrating that CVD is one of the leading causes of death and disability among patients with DM [97]. Additionally, the interaction of diabetes, especially T2DM, and CV risk factors has been shown to exacerbate mechanisms underlying vascular damage leading to atherosclerosis and heart failure [98]. Even though there has been a remarkable decline in the incidence of DM-related disorders including CVD over the last years, the relationship linking DM to CVD remains a challenge for treating DM and reducing CV events.

The epigenetic regulatory role of ncRNAs in diabetes-associated CVD is still emerging. Previous clinical studies have demonstrated that circulating lncRNAs can potentially be used to predict T2DM [42] or the outcome of heart failure [99]. With respect to the class of circRNAs, so far, the best transcript associated with T2DM and CVD is the lncANRIL (circ-ANRIL) [100]. Specifically, ANRIL levels were found to be associated with a reduced risk of atherosclerosis [27]. circRNAs have also been shown to influence increased blood glucose, inflammation, and lipid accumulation, which have a deleterious effect on blood vessels and can lead to the development of endothelial dysfunction and CVD [101]. As an example, circANKRD36 was found to be markedly upregulated in the peripheral blood cells of T2DM patients and associated with inflammatory factors [102]. This is particularly interesting since vascular inflammation is an overlapping risk factor for both diabetes and CVD. Therefore, it can be concluded that circANKRD36 may serve as a biomarker for the development of the inflammatory CVD among patients with diabetes. A previous study reported that CDR1as can regulate insulin secretion and may represent a new target for improving β-cell function in diabetes [55]. Later, another study indicated that CDR1as can function as a sponge for miR-7a to promote myocardial infarction [103]. Also more evidence demonstrated that changes in the circulating long noncoding RNAs ZFAS1 and CDR1as expression could predict acute myocardial infarction [104]. Li X et al. reported that hsa-circRNA11783-2 in human peripheral blood is correlated with CAD and T2DM [105].

**Figure 1:**
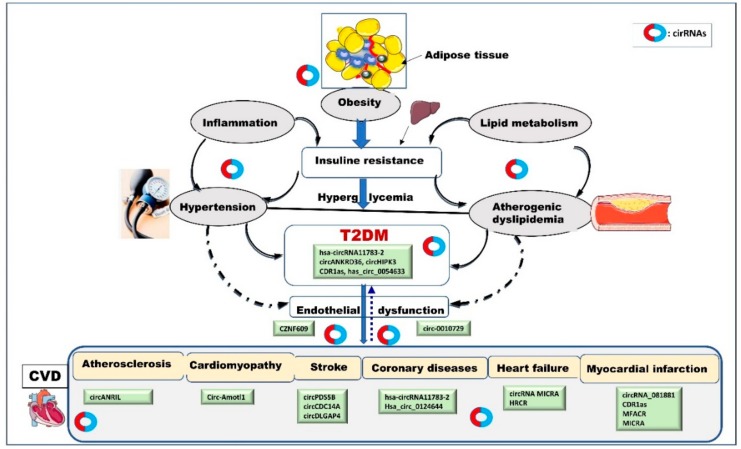
The possible pathogenic mechanisms linking diabetes to cardiovascular diseases. In addition to common genetic and environmental factors, epigenetic regulatory mechanisms such as circRNA transcripts are also postulated to play a direct or indirect role in diabetes and related cardiovascular complications. As illustrated in this figure, circRNAs may have a complex regulatory capacity in different stages of development in diabetes and CVD. Under various triggers, circRNAs (produced from local tissue or coming from the circulation) may contribute to the development of risk factors including obesity, inflammation, hypertension, hyperlipidemia, and insulin resistance, modulating diabetes progress and associated vascular disorders. circRNA transcripts may also act as a platform connecting T2DM and CVD and could be potentially used as biomarkers to predict CV outcomes in diabetes state. Cardiovascular tissues may, in turn, release circRNAs that enter a vicious cycle exacerbating diabetes, hypertension, and other diseases.

Based on these preliminary observations, it is tempting to hypothesize that circRNAs contribute to the pathophysiology of the connection between T2DM and CVD and serve as biomarkers for CVD in diabetes (Figure 1).

To the best of our knowledge, no study has yet specifically addressed the potential role of diabetes-associated circRNAs as biomarkers for the diagnosis and/or management of CVD. Nevertheless, many important yet challenging questions remain to be answered about these transcripts and their role in diabetes complications. First, from the perspective of biomarkers development, the context and the relevant dynamic network by which a specific circRNA impacts a specific CV event are not defined yet. Second, it is questionable whether a single circRNA could be used as a marker to predict complex CV outcome among patients with diabetes. Third, how circRNAs can be integrated in complex genetic networks and stress responses that regulate the full spectrum of genes expression in diabetes and its related disorders are not well defined and might represent a research area of interest. Answering these questions may aid in exploring new biomarkers for the prediction of various forms of CVD in diabetes and directing future research toward an integrated pathophysiological approach to improve understanding of these diseases that have overlapping risk factors.

## 5. Limitations and Future Perspectives

A growing number of reports suggest a role for circRNAs in the pathogenesis of diabetes and a significant utility as diagnostic and predictive biomarkers. However, numerous limitations currently hinder this field of research and delay the consideration of these RNA species for clinical settings. (i) Since circRNAs are a relatively new field of investigation, information produced from their studies are merely descriptive and correlational, and causation cannot be accurately inferred. Specifically, there is a significant lack of comprehensive knowledge about the exact origin(s), biogenesis, modulation, and regulatory circuits of circRNAs, which could impede the translation of these transcripts into the clinical setting. Thus, this challenge probably calls first for more advanced techniques in circRNAs expression quantification and functional analyses. (ii) There have been some discrepancies between studies on the dysregulation status of some circRNAs in diabetes and related complications. This is a concern particularly because studies outcomes can be influenced by several obvious factors including a lack of uniformity in study designs such as study sample size, racial/ethnic diversity, inclusion or exclusion of CVD risk factors, medication, and lifestyle. One of more of these matters could lead to erroneous and potentially misleading conclusions and therefore affect health outcomes. (iii) A significant limitation that should also be acknowledged is that the potential molecular interface connecting circRNAs in diabetes to the other CVD risk factors including hypertension, obesity, metabolic syndrome, and low-grade inflammation, is still poorly understood. Such an interface could implicate specific gene regulatory networks involving lncRNAs, miRNAs, RBPs, tissues specific transcriptional factors, and their targets. Integrating circRNAs in these likely highly orchestrated molecular networks may shed light on how these molecules may be involved in the development of diabetes and related vascular disorders, how vascular complications may, in turn, be a cause of the dysregulated circRNAs in diabetes, and how the two could be bidirectionally linked. (iv) Based on association studies, a number of authors claim that circRNA may serve as biomarkers for diabetes and CVD. However, correlation does not imply causation, and obtained preliminary results need to be clinically tested. In this respect, in vitro/in vivo validation of predicted in silico circRNA functions and targets is highly recommended as findings from the computational analysis may not reflect what happens under physiological and pathophysiological conditions. Thus, until further experimental and full clinical validation yield clean evidence of the biological role of circRNAs, the existing data should be viewed as preliminary and interpreted with caution. (v) The cell- and tissue-specific patterns of circRNAs, as well as their enhanced stability in liquid biopsy and exosomes [106], make them promising next-generation antidiabetic treatments. However, many aspects of circRNAs biology are still enigmatic. Several studies indicated that circRNAs act as miRNA sponges to control gene expression, but it is unclear whether such a mechanism can be generally considered. In addition, as with any treatment, it is anticipated that circRNAs as drugs might also cause unwanted side effects and modify drug responsiveness or cause resistance. For instance, downregulation of ANRIL lncRNA was shown to enhance cisplatin cytotoxicity via let-7a in nasopharyngeal carcinoma [107]. In the coming years, our understanding of circRNA dynamic networks may grow and new biological roles may emerge. This will lay a firmer foundation for establishing circRNAs-based therapeutic approaches and facilitate their movement from “bench to bedside”. (vi) Lately, the “metabolic memory” event has been recognized as a major impediment to the effective management of diabetic complications [108]. Epigenetic mechanisms have also been hypothesized to be a crucial interface between genetic and environmental factors to explain the metabolic memory event [108,109,110]. Interestingly, miRNAs have been shown to be implicated in the pathogenesis of metabolic memory [111], hence, the question is, are circRNAs also involved in the metabolic memory event? Exploring the potential role of these transcripts in metabolic memory could be a valuable strategy to switch-off this event and reduce its long-lasting deleterious effects of hyperglycemia on diabetic complications. Nevertheless, despite the limitations, circRNAs have immense potential as therapeutic targets and stable biomarkers for many metabolic diseases including diabetes and related disorders.

Together, the above issues and many other unanswered questions about the biology of circRNAs and their implication in diabetes remain one of the biggest challenges faced by scientists currently. Research that focuses on these issues along with the development of a multi-markers approach that combines circRNAs and clinical, genetic, epigenetic, and classical markers may help guide medical decision-making in diabetes state and hold great promise especially in terms of precision medicine.

**Table 1:** List of the main circRNA databases available online.

Database	Website	Function	References
**circBase**	http://www.circbase.org	Facilitates the identification of circRNA research in sequencing data	[112]
**circ2Traits**	http://gyanxet-beta.com/circdb	Provides a comprehensive knowledge of potential association of circRNAs with human diseases	[113]
**CircR2Disease**	http://bioinfo.snnu.edu.cn/CircR2Disease	Provides experimentally validated circRNAs associated with various diseases	[114]
**Deep Base**	http://rna.sysu.edu.cn/deepBase	Provides information on small RNAs, lncRNAs, and circRNAs from deep sequencing data	[115]
**CircInteractome**	http://circinteractome.nia.nih.gov	Explores circRNAs and their interaction with RBPs and miRNAs	[115]
**CSCD**	http://gb.whu.edu.cn/CSCD	Explores the cancer-specific circRNAs	[116]
**TSCD**	http://gb.whu.edu.cn/TSCD	Investigates tissue-specific circRNAs in the human and mouse genomes	[117]
**circBank**	http://http://www.circbank.cn	A comprehensive database for circRNAs with a standard nomenclature	[118]
**circRNADb**	http://reprod.njmu.edu.cn/circrnadb	Provides information for human circRNAs with protein-coding annotations	[119]
**circNet**	http://circnet.mbc.nctu.edu.tw	Provides circRNA-miRNA gene controlling networks	[120]
**starBase v2.0**	http://starbase.sysu.edu.cn	Identifies the RNA-RNA and protein-RNA interaction networks from large-scale CLIP-Seq data	[121]
**circlncRNAnet**	http://app.cgu.edu.tw/circlnc	Provides a “one-stop” resource for in-depth analyses of circRNA/ncRNA biology	[122]
**exoRBase**	http://www.exoRBase.org	Provides information on circRNAs, lncRNAs and miRNAs in human blood exosomes	[123]
**TRCirc**	http://www.licpathway.net/TRCirc	Provides a resource for transcriptional regulatory information of circRNAs	[124]
**CircFunBase**	http://bis.zju.edu.cn/CircFunBase	Provides high-quality functional circRNAs resource	[125]
**CIRCpedia v2**	http://www.picb.ac.cn/rnomics/circpedia	Contains comprehensive circRNA annotations and allows expression comparison in tissues	[126]

Abbreviations: circRNAs, circular RNAs; miRNAs, microRNAs; lncRNAs, long noncoding RNAs; RBP, RNA binding protein.

**Table 2:** Putative functions of relevant circular RNAs in diabetes mellitus and associated vascular complications.

Circular RNA	Expression	Potential Function and Phenotype	References
**Diabetes/Glucose** **Homeostasis/CVD**			
CDR1as/cirRS-7	↑	Improves insulin secretion and transcription through inhibiting miR-7 and accelerating Myrip and Pax6 expression	[55]
circRNA-HIPK3	↑	Regulates islet cell function by sequestering miR-124–3p and miR-338–3p and elevating Slc2a2, Akt1 and Mtpn	[57]
hsa_circ_0054633	↑	Potential diagnostic biomarker of pre-diabetes and T2DM in peripheral blood cells	[50]
circRNA-WDR77	↑	Regulates proliferation and migration of high glucose-induced VSMCs by affecting the expression of FGF-2 through miR-124 sponging	[52]
circANKRD36	↑	Potential biomarker for screening chronic inflammation in patients with T2DM	[102]
**Diabetic Cardiomyopathy**			
circRNA_000203	↑	Exacerbates myocardial fibrosis in mouse cardiac fibroblasts via inhibiting the interaction of miR-26b-5p with the target genes	[94]
circRNA_010567	↑	Promotes the development of diabetic cardiomyopathy through the circRNA_010567/miR-141/TGF-β1 axis	[95]
hsa-circ-0076631 (CACR)	↑	Mediates pyroptosis of diabetic cardiomyopathy by functioning as miR-214-3p sponge	[96]
**Diabetic Nephropathy**			
cirRNA_15698	↑	circRNA_15698/miR-185/TGF-β1 axis promoted extracellular matrix (ECM)-related protein synthesis in diabetic nephropathy progression	[71]
**Gestational Diabetes**			
circ_5824, circ_3636, circ_0395	↓	Suspected to be involved in the occurrence and pathogenesis of GDM	[79]
hsa-circRNA_0054633	↑	Change in its expression in the placental villi of GDM patients may reflect its potential role in the development of GDM	[78]
**Diabetic Retinopathy**			
circRNA-0005015	↑	Involved in diabetes retinopathy by acting as miR-519d-3p sponge to increase the expression of its target genes, MMP-2, XIAP, and STAT3	[64]
circRNA-HIPK3	↑	Promotes retinal vascular disorders by blocking miR-30a-3p members function to reverse the expression of their target genes VEGF, FZD4, and WNT2	[65]
cZNF609	↑	Role in mediating vascular dysfunction by acting as miR-615-5p sponge	[66]
circRNA-cPWWP2A	↑	Alleviates diabetes mellitus-induced retinal vascular dysfunction by sponging miR-579	[67]

Abbreviations: CVD, cardiovascular disease; FZD4, frizzled class receptor 4; GDM, gestational diabetes mellitus; MYRIP, myosin VIIA and Rab interacting protein; MMP-2, matrix metalloproteinase-2; PAX6, the paired box gene 6; SLC2A2, solute carrier family 2 member 2; STAT3, signal transducer and activator of transcription 3; TGF-β, transforming growth factor-beta; VEGF, vascular endothelial growth factor; T2DM, type 2 diabetes mellitus; VSMCs, vascular smooth muscle cells; WNT2, Wingless-type MMTV integration site family, member 2; XIAP, X-linked inhibitor of apoptosis protein; ↑, up-regulated; ↓, down-regulated.
